# The Role of Systemic Therapy in Resectable Colorectal Liver Metastases: Systematic Review and Network Meta-Analysis

**DOI:** 10.1093/oncolo/oyac212

**Published:** 2022-10-14

**Authors:** Mohamad Bassam Sonbol, Rabbia Siddiqi, Pedro Luiz Serrano Uson, Surabhi Pathak, Belal Firwana, Gehan Botrus, Diana Almader-Douglas, Daniel H Ahn, Mitesh J Borad, Jason Starr, Jeremy Jones, Chee-Chee Stucky, Rory Smoot, Irbaz Bin Riaz, Tanios Bekaii-Saab

**Affiliations:** Department of Oncology Mayo Clinic Cancer Center, Phoenix, AZ, USA; Department of Medicine, Dow University of Health Sciences, Karachi city, Sindh, Pakistan; Department of Oncology Mayo Clinic Cancer Center, Phoenix, AZ, USA; Department of Oncology, Hospital Israelita Albert Einstein, São Paulo, Brazil; King’s Daughters Medical Center, Ashland, KY, USA; Heartland Cancer Research, Missouri Baptist Medical Center, St Louis, MO, USA; Department of Medicine, Honorhealth Research Institute, Scottsdale, AZ, USA; Mayo Clinic Libraries, Mayo Clinic, Phoenix, AZ, USA; Department of Oncology Mayo Clinic Cancer Center, Phoenix, AZ, USA; Department of Oncology Mayo Clinic Cancer Center, Phoenix, AZ, USA; Mayo Clinic Cancer Center, Jacksonville, FL, USA; Mayo Clinic Cancer Center, Jacksonville, FL, USA; Department of Oncology Mayo Clinic Cancer Center, Phoenix, AZ, USA; Mayo Clinic Cancer Center, Rochester, MN, USA; Department of Oncology Mayo Clinic Cancer Center, Phoenix, AZ, USA; Mayo Clinic Cancer Center, Rochester, MN, USA; Department of Oncology Mayo Clinic Cancer Center, Phoenix, AZ, USA

## Abstract

**Background:**

Despite multiple randomized trials, the role of perioperative chemotherapy in colorectal cancer liver metastasis (CRLM) is still under debate. In this systematic review and network meta-analysis (NMA), we aim to evaluate the efficacy of perioperative systemic therapies for patients with CRLM.

**Methods:**

We searched various databases for abstracts and full-text articles published from database inception through May 2021.We included randomized controlled trials (RCTs) comparing the addition of perioperative (post, pre, or both) systemic therapies to surgery alone in patients with CRLM. The outcomes were compared according to the chemotherapy regimen using a random effects model. Outcomes of interest included disease-free survival (DFS) and overall survival (OS).

**Results:**

Seven RCTs with a total of 1504 patients with CRLM were included. Six studies included post-operative treatment and one evaluated perioperative (pre- and postoperative) therapy. Fluoropyrimidine-based chemotherapy was the most used systemic therapy. NMA showed benefit of adding perioperative therapy to surgery in terms of DFS (HR 0.73, 95% CI 0.63 to 0.84). However, these findings did not translate into a statistically significant OS benefit (HR 0.88, 95% CI 0.74 to 1.05). NMA did not show any advantage of one regimen over another including oxaliplatin or irinotecan.

**Conclusions:**

This systematic review and NMA of 7 RCTs found that the addition of perioperative systemic treatment for resectable CRLM could improve disease-free survival but not overall survival. Based on the findings, addition of perioperative treatment in resectable CRLM should be individualized weighing the risks and benefits.

## Teaser

The role of perioperative chemotherapy in colorectal cancer liver metastasis is unclear. This review evaluates the efficacy of perioperative systemic therapies for patients with colorectal cancer liver metastasis.

**Implications for Practice**

The role of adding systemic therapy to surgery in patients with resectable colorectal liver metastases is unclear. In this network meta-analysis of 7 trials, we found that the addition of systemic therapy improves disease-free survival but not overall survival. Therefore, chemotherapy should not be uniformly recommended in this setting.

## Introduction

The liver is the most common site of metastases in patients with colorectal cancer (CRC) and accounts for at least two-thirds of all CRC deaths.^1^ Overall, 25% of patients with CRC present with colorectal liver metastasis (CRLM) and 30-60% of patients develop CRLM. While the goal of treatment in metastatic colorectal cancer is generally palliative, long remission has been reported in patients with resectable CRLM after surgery with resectable patient having significantly better survival than unresectable.^2,3^

However, the role of perioperative systemic therapy in such patients is less clear. Multiple studies have been conducted over the last 2 decades investigating the role of perioperative systemic therapy along with which regimen to be used with variable results.^4-13^ Therefore, A network meta-analysis (NMA) is helpful to compare different agents across randomized clinical trials (RCTs) and delineate the actual role of such therapies, especially with the recent JCOG0603 study which showed discrepancy between overall- and disease-free survival outcomes.^14^ This is especially important because current guidelines list the approaches available with no guidance regarding which strategy is preferred.^15^ In this systematic review and network meta-analysis, we aim to evaluate the efficacy of perioperative systemic therapies for patients with resectable CRLM.

## Methods

The reporting of this systematic review follows the Preferred Reporting Items for Systematic Review and Meta-Analyses (PRISMA) statement.^16^

### Study Objective

The current study aimed to evaluate the efficacy of perioperative systemic therapy addition to surgery in patients with resectable CRLM.

### Eligibility Criteria

Randomized phases II and III controlled trials were included if they were published in English-language. Trials of interest were comparing surgery alone with perioperative (post, pre, or both) systemic therapy in patients with resectable CRLM. Definition of resectable CRLM was per the study of interest. Studies evaluating the efficacy of epidermal growth factor receptor antibodies were excluded as patients included in such studies have better prognosis making transitivity assumption not possible.

### Data Sources and Search Strategies

A comprehensive literature search was performed for abstracts and full text articles published in print or online from database inception up through May 2021 from electronic databases, MEDLINE, EMBASE, Scopus, Web of Science, and the Cochrane Central Register of Controlled Trials (CENTRAL).

Detailed search strategy is as described in (Supplementary Methods). The search strategy was designed and conducted by an experienced librarian with input from the study investigators. Outcomes of interests included: overall-survival (OS) and disease-free survival (DFS).

### Study Selection

Two individual reviewers (M.B.S. and P.L.J.) identified and reviewed full-text articles and abstracts that were deemed relevant by screening the list of titles. Disagreements between the two reviewers were resolved with consensus.

### Data Extraction

Prespecified data elements were extracted from each trial utilizing a structured data abstraction form, including baseline characteristics, sample size, and interventions used. Two reviewers extracted the data from the included trials (S.P. and G.B.), and disagreements were resolved by referring to a third reviewer (M.B.S.).

### Risk of Bias and Certainty of Evidence

The Cochrane Collaboration’s tool for assessing the risk of bias in the trial was used,^17^ which includes the following domains: random sequence generation, allocation concealment, blinding, incomplete outcome data, and selective outcome reporting. Two reviewers independently assessed trial quality (B.F. and M.B.S.) and disagreements were resolved with consensus. The certainty of evidence (ie, certainty in the estimates) was evaluated using the GRADE approach (Grading of Recommendations Assessment, Development, and Evaluation).^18^

### Statistical Analysis

The statistical analysis was conducted using R statistical software version 3.6.3 (R Project for Statistical Computing). Pre-calculated hazard ratios (HR) with corresponding 95% CIs, abstracted from the included trials, were log transformed and then pooled together. Mixed treatment comparisons were made using a fixed-effects network meta-analysis within the frequentist framework.^19^ League tables and forest plots were generated for back-transformed network estimates. Heterogeneity between and within designs was assessed using Cochran’s Q and quantified using *I*^2^ statistics. *I*^2^ values of <25%, 25%-75% and > 75% represented low, moderate, and high degree of heterogeneity, respectively. *P*-scores, which are frequentist analogs to surface under the cumulative ranking curve (SUCRA),^20^ were computed and used to rank treatments. Rankograms were made to visualize treatment ranking based on *P*-scores. A higher *P*-score indicated a better efficacy. The NMA was carried out using R package “netmeta” (version 1.2.0).

## Results

### Study Selection and Characteristics

**Figure 1.**
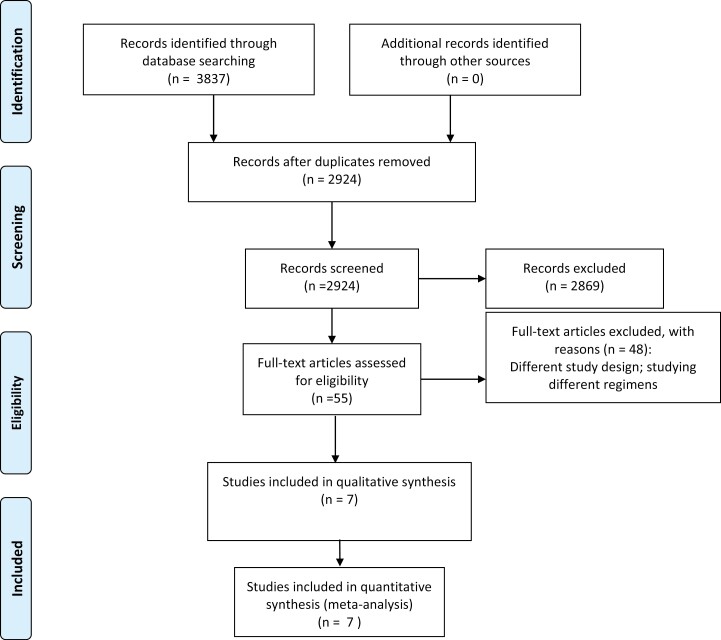
PRISMA flow diagram showing screening and selection process.

A total of 2924 titles and abstracts were identified by the screening electronic search strategy, of which 55 met the eligibility for assessment (Fig. 1). Seven references were identified reporting 7 studies.^4-14^ The 7 studies encompassed a total of 1504 patients with metastatic colorectal cancer (mCRC) (Supplementary Table S1). Six studies included post-operative treatment and one evaluated perioperative (pre- and postoperative) therapy. Surgery was the control arm in 5 trials.^4,7,9,12^ Fluoropyrimidine monotherapy (FP) was the most used (4 trials) systemic therapy, followed by (fluoropyrimidine plus oxaliplatin) FPOX (3 trials), (infusional 5-Fluorouracil + irinotecan) FOLFIRI (one trial), and capecitabine plus oxaliplatin (CapeOx) and bevacizumab (one trial) (Supplementary Fig. S1 and Table S1).

Age in these trials ranged from 20 to 82 years. Disease (recurrence)-free survival was the primary end point in all studies except one where OS was the primary end point.^12^ Slow accrual was noted in most trials leading to early study termination in 5 trials.^7,8,12,13,21^ Most of the trials included patients with synchronous and metachronous liver metastases. The characteristics of the included RCTs are outlined in Supplementary Table S1.

### Is There Any Benefit of Any Perioperative Systemic Therapy?

**Figure 2.**
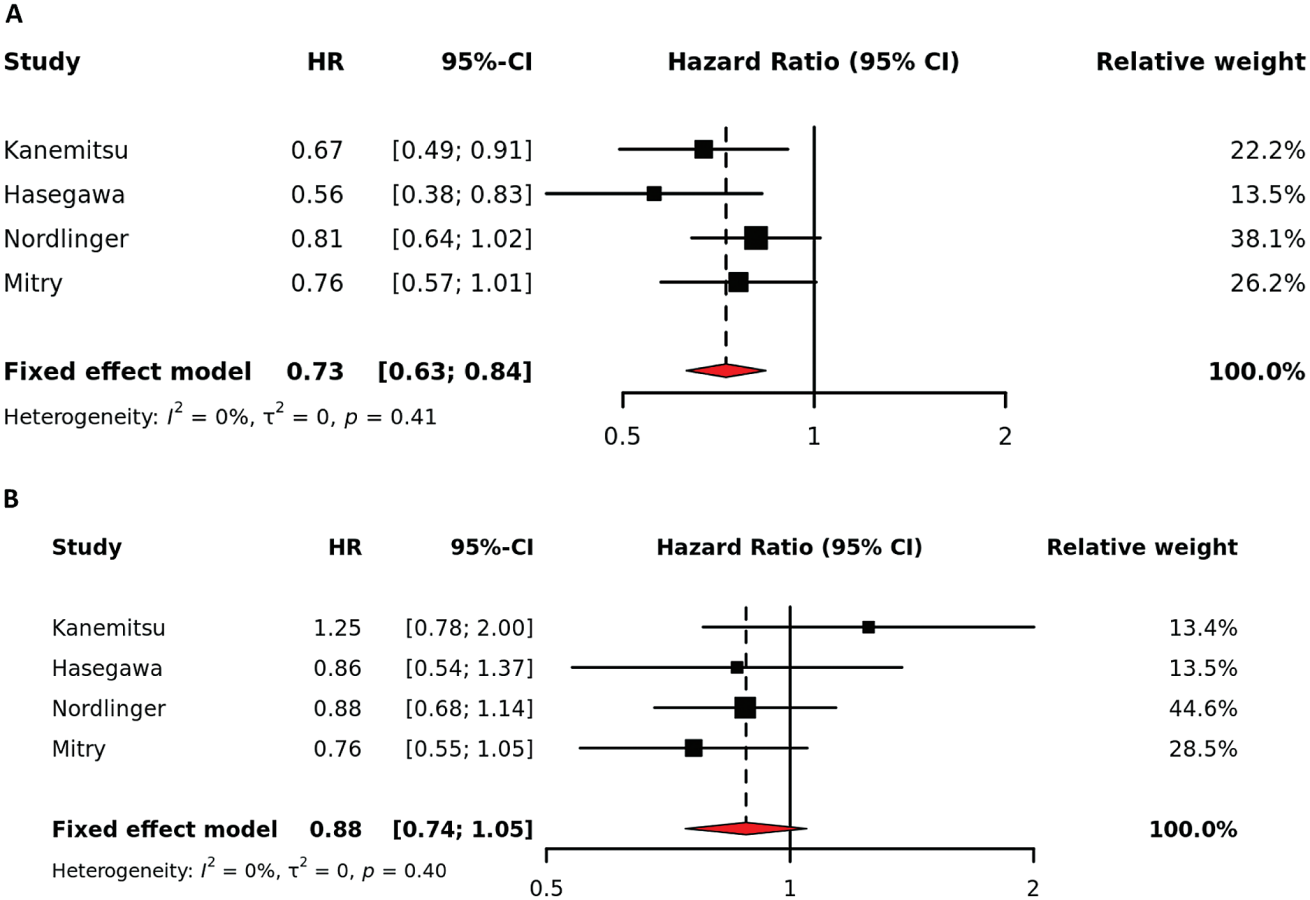
Meta-analysis of the included studies examining disease-free survival (panel A) and overall survival (panel B) for perioperative treatment vs. observation. The marker size indicates the relative weight of the study as it contributes to the results of the overall comparison. HR indicates hazard ratio.

Five studies examined the role of perioperative treatment compared to surgery alone.^4,7,9,12^ FPOX was used in 2 studies and FP monotherapy in one (Supplementary Table S1). The pooled direct comparisons showed a benefit of adding perioperative therapy to surgery in terms of DFS (HR 0.73, 95% CI 0.63 to 0.84). However, these findings did not translate into a statistically significant OS benefit (HR 0.88, 95% CI 0.74 to 1.05) (Fig. 2). These results are consistent with the results of the included trials where a DFS (or trend for OS) benefit was seen with perioperative treatment vs. surgery alone except for Kanemitsu et al.^7,14^ In the JCOG0603 study (Kanemitsu et al), postoperative chemotherapy with infusional fluorouracil plus oxaliplatin (FOLFOX) improved DFS but worsened OS over surgery alone.

### Which Perioperative Therapy?

In the network meta-analysis, we found that any of the perioperative regimens (FP, FPOX, or FOLFIRI) led to statistically significant DFS benefit compared to surgery alone except for CapeOx plus bevacizumab (HR 0.79, 95% CI 0.42 to 1.47) (Table 1). However, none of these regimens led to a statistically significant OS benefit.

**Table 1.**
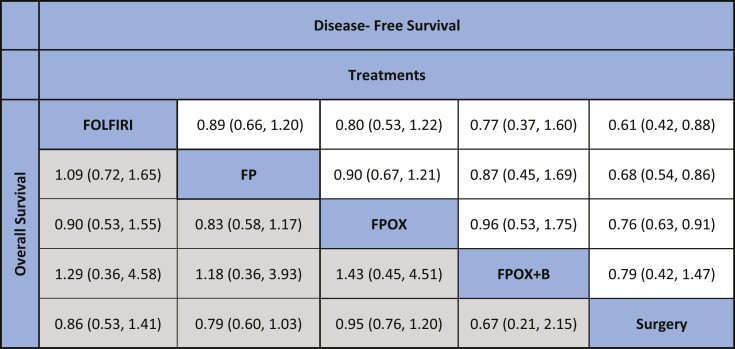
League table showing indirect comparisons among first-line treatments.

Hazard ratios (HRs) and 95% CIs for the pairwise comparisons of the network meta-analysis from indirect comparisons. Comparisons should be read from left to right. The HRs for comparisons are in the cell in common between the column-defining and row-defining treatment. For progression-free survival, an HR of less than 1 favors row-defining treatment. For overall survival, an HR of less than 1 favors column-defining treatment.

Abbreviations: FP, fluoropyrimidine; FPOX, fluoropyrimidine and oxaliplatin; FPOX+B, FPOX and bevacizumab.

In addition, when comparing those perioperative regimens to each other, the network meta-analysis shows that FOLFIRI or FP were ranked the highest in DFS (*P* score of 84% and 66% for FOLFIRI and FP, respectively) and FP and CapeOx plus bevacizumab were ranked the highest in OS (*P* score of 72% and 68% for FP and CapeOx plus bevacizumab, respectively). (Table 1; Supplementary Figs. S2 and S3). Making a conclusion of which regimen is superior in terms of ranking (P score) is not possible given that NMA did not show any advantage of one regimen over another.

### Risk of Bias

A qualitative assessment was performed by assessing various indicators for each individual study using the Cochrane tool for risk of bias. Overall, the trials were deemed to be at low risk of bias except for detection bias for the outcome of DFS given that most of the trials were not blinded. (Supplementary Fig. S4).

### Certainty of Evidence

The certainty of the evidence for the DFS in both direct and indirect comparisons for the different perioperative chemotherapy regimens studied was high, except for the CapeOx-B regimen which was moderate due to imprecision. The certainty of the evidence for the OS in both direct and indirect comparisons for the different perioperative chemotherapy regimens studied was moderate due to imprecision (Supplementary Table S2).

## Discussion

In this systematic review and network meta-analysis of patients with resectable CRLM, we found that the addition perioperative chemotherapy to surgery only improves DFS without OS benefit.

While the overall goal of treatment in mCRC is palliative, long-term remission is possible in subset of patients with resectable oligometastatic CRLM.^2,3,22-24^ Therefore, identifying such patients is important with plan of care involving multidisciplinary team approach. Surgery on the primary and oligometastatic disease has been the standard of care but the benefit of adding perioperative systemic therapy have been questioned in multiple studies with mostly DFS, but no OS, benefit. The main criticism of such studies is the small sample size and being powered to only detect DFS benefit rather than OS. In our network meta-analysis, we found that perioperative systemic therapy was associated only with improved DFS but not OS. This is consistent with most of the individual studies included in our analysis except for Kanemitsu et al (JCOG0603)^7^ where adjuvant FOLFOX was associated with numerically worse OS compared to surgery alone. JCOG0603 randomized patients between observation or chemotherapy with 6 months of FOLFOX after surgery and was terminated early as FOLFOX demonstrated improved DFS but worse OS. The lack of survival benefits unclear but can potentially be explained by multiple hypotheses. First, DFS can be sometimes overestimated due to the challenges in detecting recurrences in liver given the post-oxaliplatin changes in liver parenchyma based on sinusoidal obstruction syndrome.^25^ This is also important as older studies such as Nordlinger et al had ultrasound as an option for surveillance instead mandating advanced imaging such as computerized tomography.^9,10^ However, it is unclear whether MRI would be feasible and add any benefit in surveillance of such patients given its superiority in identifying CRLM especially in those treated with chemotherapy.^26,27^ Theoretically, detecting liver recurrences early on can lead to earlier salvage surgical resection in such patients which could affect overall survival. Second, the use of oxaliplatin in the adjuvant setting along with its associated toxicity (grade 3/4 10%) restricted its use in the chemo arm upon recurrence (64% vs. 44% in surgery and chemo arm, respectively). Third, it is possible that the inferior OS could reflect a statistical artifact given the small number of events at the time of the analysis. Therefore, long-term follow up is needed.^28^

Timing of perioperative systemic therapy is also important. While most of the studies included in our analysis (except Nordlinger et al) used post-operative therapy, there is an increase interest in moving systemic therapy upfront. Neoadjuvant therapy has several theoretical advantages such as downsizing tumors preoperatively along with increasing R0 resection rates and testing tumor sensitivity to chemotherapy.^29^ However, chemotherapy has been associated with increased risk of steatosis, steatohepatitis, and sinusoidal injury. In addition to the sinusoidal obstruction syndrome seen from oxaliplatin, irinotecan exposure can lead to steatohepatitis which is associated with increased 90-day mortality. Therefore, identifying patients who would derive most of the benefit from systemic therapy is vital. For example, in a multivariate analysis of EORTC 40983, perioperative FOLFOX was found to mostly benefit patients with elevated CEA and better performance status regardless of the number of metastatic lesions.^30^ Future studies can potentially integrate circulating tumor DNA as predictive biomarker for chemotherapy benefit in patients with CRLM.^31,32^

The choice of systemic therapy in the perioperative setting is also important. Most of the studies included in our analysis utilized fluoropyrimidine monotherapy or combined with oxaliplatin with only one using FOLFIRI. Bevacizumab was only used in one study. In our NMA, all the regimens showed improved in DFS compared to surgery except for the CapeOx plus bevacizumab. Making a conclusion of which regimen is superior in terms of ranking (P score) is not possible given that NMA did not show any advantage of one regimen over another. More recently, multiple studies have shown the improved outcomes using more aggressive chemotherapy with triplet backbone regimen (FOLFOXIRI) compared to doublets (FOLFOX or FOLFIRI) in mCRC.^33,34^ A recent pooled analysis of TRIBE1 and TRIBE2 studies showed that the benefit of FOLFOXIRI/bevacizumab was regardless of tumor burden and sustained in those with oligometastatic disease.^35^ Whether more aggressive chemotherapy with triplet would add benefit beyond surgery is not clear. Similarly, the role of monoclonal antibody, targeting vascular endothelial grown factor (VEGF) or epidermal growth factor receptor (EGFR) is not established. In fact, the addition of cetuximab to chemotherapy (compared to chemotherapy alone) in the perioperative setting showed worse PFS and OS in the new EPOC study.^36^ Conversely, the PARLIM (AIO KRK 0314) phase II study showed contrasting findings. The addition of panitumumab to 12 weeks of postoperative FOLFOX in resected oligometastatic CRC showed more favorable outcome in terms of PFS and OS. However, given the negative phase III data (NEW EPOC), the addition of anti-EGFR monoclonal antibody in oligometastatic CRC undergoing resection is discouraged. On the other hand, caution should be made in the interpretation of these studies, considering that a full genomic analysis was not performed in both. Patients were accrued just by limited KRAS status wild type, considering that NRAS, HRAS, BRAF, Her-2, and MSI-H status could directly influence the efficacy of anti-EGFR monoclonal antibodies and disease specific outcomes.^37^

The limitations to this study are related to the nature of this network analysis because some of evidence was driven from indirect comparisons. In addition, this analysis was performed with study-level data rather than individual patient data, which would limit the power of the analysis. Furthermore, studies evaluating the efficacy of epidermal growth factor receptor antibodies were excluded as patients included in such studies have better prognosis making transitivity assumption not possible. Despite these limitations, we believe that this NMA allows a better understanding of the current role of systemic therapy in resectable CRLM with including most recent RCTs in this analysis.

## Conclusions

The mainstay of treatment for patients with resectable CRLM is surgery. The addition of chemotherapy in the perioperative setting should be individualized with weighing the benefit (only DFS) and risks with the toxicities associated with treatment.

## Supplementary Material

oyac212_suppl_Supplementary_Material

## Data Availability

The data underlying this article will be shared on reasonable request to the corresponding author.
