# Supplementary material for: The Role of Systemic Therapy in Resectable Colorectal Liver Metastases: Systematic Review and Network Meta-Analysis
Source: Oncologist. 2022 Oct 14;27(12):1034–40. doi: 10.1093/oncolo/oyac212 (PMC9732220; doi:10.1093/oncolo/oyac212)
Supplement: oyac212_suppl_Supplementary_Material [file oyac212_suppl_supplementary_material.docx]

# eMethods: Search Strategy:

**8.20.20 PubMed (1660)** (((((((("Colorectal Neoplasms"[Mesh]) AND "Liver Neoplasms"[Mesh]) AND "Neoplasm Metastasis"[Mesh]) OR (resect* colorectal liver metast*)) OR (colorectal cancer [ti] OR CRC [tiab] OR mCRC [tiab] OR CLM [tiab])) OR (oligometasta*)) AND (chemo* [ti] OR resect* [ti] OR surgic* [ti] OR surger* [ti] OR hepatectom* [ti]))) AND (survival) AND ((clinicaltrial[Filter] OR clinicaltrialphaseii[Filter] OR clinicaltrialphaseiii[Filter] OR comparativestudy[Filter] OR meta-analysis[Filter] OR randomizedcontrolledtrial[Filter] OR systematicreview[Filter]) AND (english[Filter])) Filters: Clinical Trial, Clinical Trial, Phase II, Clinical Trial, Phase III, Comparative Study, Meta-Analysis, Randomized Controlled Trial, Systematic Review, English

**8.24.20 Embase <1974 to 2020 August 21> Search Strategy:**

1 exp colorectal cancer/ (181721)

2 exp liver cancer/ (247755)

3 exp metastasis/ (633181)

4 exp colorectal liver metastasis/ (4532)

5 resect* colorectal liver metast*.mp. (407)

6 colorectal cancer.ti. (88518)

7 (CRC or mCRC or CLM).ti. or (CRC or mCRC or CLM).ab. (61663)

8 oligometasta*.mp. (3874)

9 (chemo* or resect* or surgic* or surger* or hepatectom* or metastas* or metastat*).ti. (1270145)

10 survival.mp. (1728324)

11 exp comparative study/ (1417059)

12 exp phase 2 clinical trial/ (82223)

13 exp phase 3 clinical trial/ (47965)

14 exp randomized controlled trial/ (617472)

15 exp "systematic review"/ (257745)

16 exp meta analysis/ (194284)

17 1 and 2 and 3 (15909)

18 4 or 5 or 17 (19249)

19 6 or 7 or 8 (115100)

20 18 and 19 (8684)

21 9 and 10 and 20 (4301)

22 exp clinical trial/ (1515430)

23 11 or 12 or 13 or 14 or 15 or 16 or 22 (3037741)

24 21 and 23 (1082)

25 limit 24 to english language (1059)

**8.31.20 Scopus (549)** ( ( ( ( TITLE-ABS-KEY ( "resectable colorectal liver metasta*" ) OR TITLE-ABS-KEY ( "resectable cancer liver metasta*" ) ) ) OR ( ( ( ( TITLE ( "colorectal cancer" OR clm OR mcrc OR crc ) OR ABS ( "colorectal cancer" OR clm OR mcrc OR crc ) ) ) AND ( ( TITLE ( metasta* OR resect* OR advanced ) OR ABS ( metasta* OR resect* OR advanced ) ) ) ) AND ( ( TITLE ( resect* OR surgic* OR surger* OR chemo* OR hepatectom* ) OR ABS ( resect* OR surgic* OR surger* OR chemo* OR hepatectom* ) ) ) ) ) AND ( ( TITLE ( "clinical trial phase II" OR "clinical trial phase 2" OR "clinical trial phase III" OR "clinical trial phase 3" OR "systematic review" OR "meta analysis" OR "comparative study" ) OR ABS ( "clinical trial phase II" OR "clinical trial phase 2" OR "clinical trial phase III" OR "clinical trial phase 3" OR "systematic review" OR "meta analysis" OR "comparative study" ) ) ) ) AND ( TITLE-ABS-KEY ( survival ) ) AND ( LIMIT-TO ( LANGUAGE , "English" ) )

**8.31.20 Web of Science (549)**

TITLE: ("colorectal cancer" OR clm OR mcrc OR crc) or AB=("colorectal cancer" OR clm OR mcrc OR crc) and TI=(metasta* or resect* or advanced) or AB=(metasta* or resect* or advanced)

Or TS=("resectable colorectal liver metasta*" OR "resectable cancer liver metasta*") AND

AB=(resect* OR surgic* OR suger* OR chemo* OR hepatectom* ) or TI=(resect* OR surgic* OR suger* OR chemo* OR hepatectom* ) AND AB=("clinical trial phase II" OR "clinical trial phase 2" OR "random* clinical trial*" OR "clinical trial phase III" OR "clinical trial phase 3") or TI=("clinical trial phase II" OR "clinical trial phase 2" OR "random* clinical trial*" OR "clinical trial phase III" OR "clinical trial phase 3") =("systematic review" OR "meta analysis" OR "comparative study") or TI=("systematic review" OR "meta analysis" OR "comparative study") and TOPIC: (survival) Refined by: LANGUAGES: ( ENGLISH ) Indexes=SCI-EXPANDED, ESCI Timespan=All years

**8.31.20 EBM Reviews - Cochrane Database of Systematic Reviews <2005 to August 26, 2020>**

**Search Strategy:**

1 advanced colorectal cancer.mp. [mp=title, abstract, full text, keywords, caption text] (11)

2 liver metasta*.mp. [mp=title, abstract, full text, keywords, caption text] (49)

3 oligometas*.mp. [mp=title, abstract, full text, keywords, caption text] (6)

4 resectable colorectal.mp. [mp=title, abstract, full text, keywords, caption text] (2)

5 (surgic* or surger* or chemo* or hepatectom*).mp. [mp=title, abstract, full text, keywords, caption text] (5262)

6 (metasta* or resect* or advanced).mp. [mp=title, abstract, full text, keywords, caption text] (2600)

7 (CRC or mCRC or CLM).mp. [mp=title, abstract, full text, keywords, caption text] (65)

8 6 and 7 (46)

9 1 or 8 (52)

10 2 or 3 or 4 (55)

11 9 and 10 (12)

12 5 and 11 (12)

13 survival.mp. [mp=title, short title, abstract, full text, keywords, caption text] (2634)

14 12 and 13 (11)

**8.13.20 Author supplied (9)**

| DATABASE | RESULTS | DUPLICATES | REMAINING |
| --- | --- | --- | --- |
| PubMed | 1660 | 109 | 1551 |
| Embase | 1059 | 196 | 863 |
| Scopus | 549 | 313 | 236 |
| Web of Science | 549 | 287 | 262 |
| CDBSR | 11 | 3 | 8 |
| Author Supplied | 9 | 5 | 4 |
| **TOTAL** | 3837 | 913 | 2924 |

eFigure1


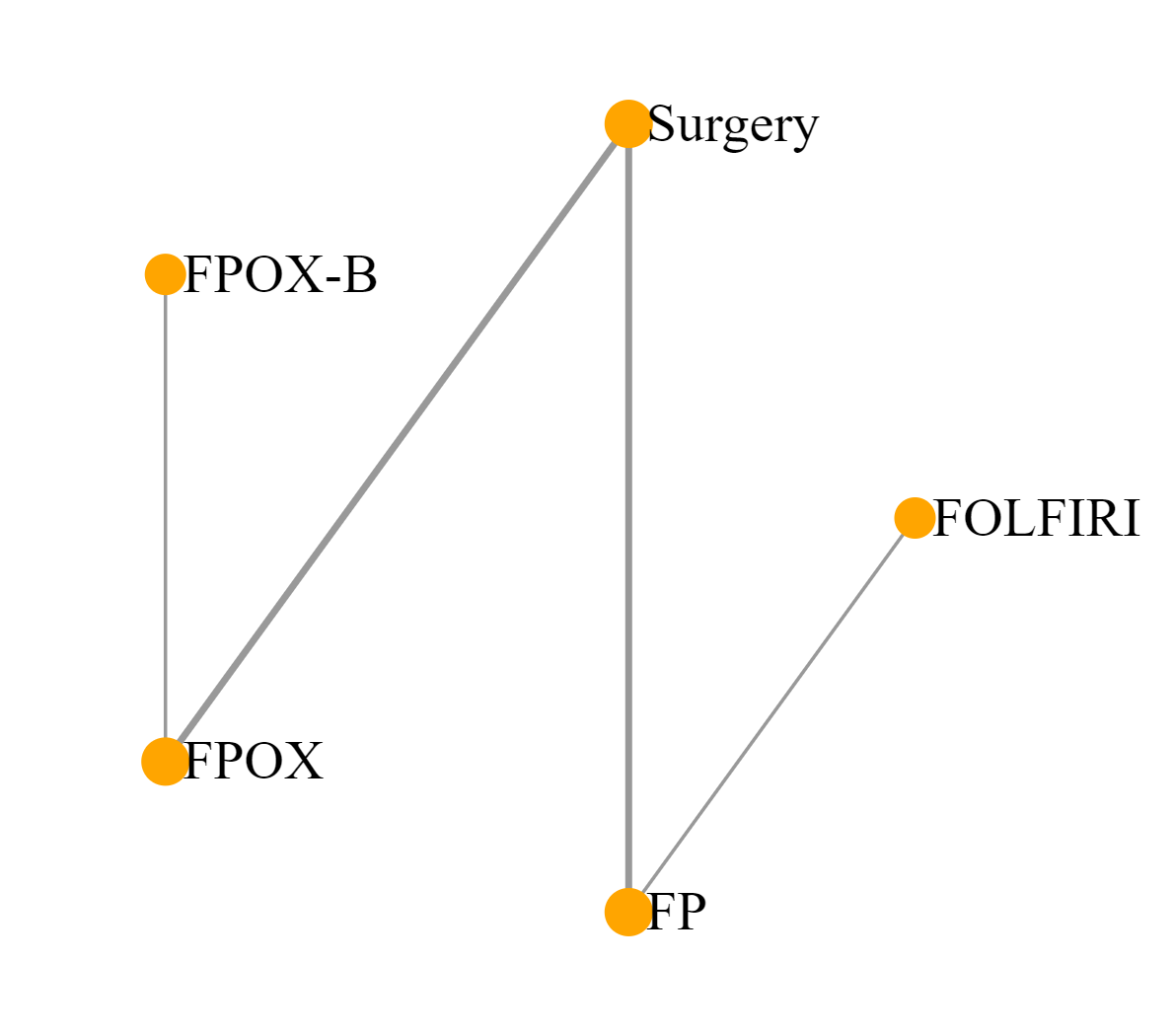


Legend: FPOX: fluoropyrimidine + oxaliplatin, FP: fluoropyrimidine alone, B: bevacizumab, FOLFIRI: infusional fluorouracil + irinotecan

|  | **Arm** | **n** | **End Points** | **Median Age** | **Synchronous liver mets** | **# liver mets** | **Maximum size of liver mets** |  |
| --- | --- | --- | --- | --- | --- | --- | --- | --- |
| Kanemitsu 2020 | S | 149 | DFS (1), OS (2) | 65 (44-75) | 56% | 1-3 (91%), 4- (9%) | < 5 cm (87%), ≥ 5 cm (13%) |  |
|  | S+FOLFOX | 151 |  | 63 (27-74) | 56% | 1-3 (90%), 4- (10%) | < 5 cm (85%), ≥ 5 cm (15%) |  |
|  |  |  |  |  |  |  |  |  |
|  |  |  |  |  |  |  |  |  |
| Saiura 2014 | S | 90 | RFS(1); OS(2) | 64.4(9.2) | 44.9% | 1(49.4%), >1 (50.6%) | <5 cm (79.7%), >5cm (20.3%) |  |
|  | S+ UFT/LV | 90 |  | 62.3(8.5) | 44.3% | 1(42%), >1(58%) | <5cm (76.1%), >5cm (23.9%) |  |
|  |  |  |  |  |  |  |  |  |
| Snoeren 2017 | S+CapeOx | 39 | DFS (1); OS(2) | 61 (53-63) | 49% | 1-4 (82%), >/=4(18%) | NR |  |
|  | S+CapeOx+Bev | 40 |  | 62 (57-70) | 50% | 1-4(80%), >/=4(20%) |  |  |
|  |  |  |  |  |  |  |  |  |
|  |  |  |  |  |  |  |  |  |
| Nordlinger 2013 | S | 182 | PFS(1),OS(2) | 64(25-78) | 37% | 1-3(92%),4(8%),>4(1%) | NR |  |
|  | FOLFOX-S-FOLFOX | 182 |  | 62(29-79) | 34% | 1-3(93%),4(7%) |  |  |
|  |  |  |  |  |  |  |  |  |
| Ychou 2009 | S+5FU/LV | 160 | DFS(1), OS(2) | 61(34-76) | 62.30% | 1-4(97.4%),>/=4(2.6%) | NR | |
|  | S+FOLFIRI | 161 |  | 63(27-75) | 61.40% | 1-4(96.7%),>/=4(3.3%) |  |  |
|  |  |  |  |  |  |  |  |  |
| portier 2006 | S | 85 | DFS(1),OS (2) | 63(36-76) | 29.40% | 1-3(95.3%),>/=4(4.7%) | <5 cm (69.4%),>5 cm (30.6%) |  |
|  | S+5-FU/LV | 86 |  | 63(35-77) | 27.90% | 1-3(95.3%),>/=4(4.7%) | <5 cm (74.4%),>5 cm (25.6%) |  |
|  |  |  |  |  |  |  |  |  |
|  |  |  |  |  |  |  |  |  |
| Langer 2002/Mitry 2008 | S | 55 | OS(1)DFS(2) | 60(20-82) | NA | 1(67.3%),>/=2(32.7%) | NR |  |
|  | S+5-FU/LV | 52 |  | 63.5(35-76) | Yes(32.7%),No(65.4%) | 1(63.5%),>/=2(36.5%) |  |  |

eTable1

Legend: FOLFIRI: infusional fluorouracil + irinotecan, CapeOx: Capecitabine + Oxaliplatin, FOLFOX: infusional fluorouracil + oxaliplatin, S: surgery, 5-FU/LV: infusional fluorouracil + leucovorin, UFT/LV: uracil tegafur + leucovorin, Bev: bevacizumab

eFigure 2: : Ranking of interventions for disease-free survival


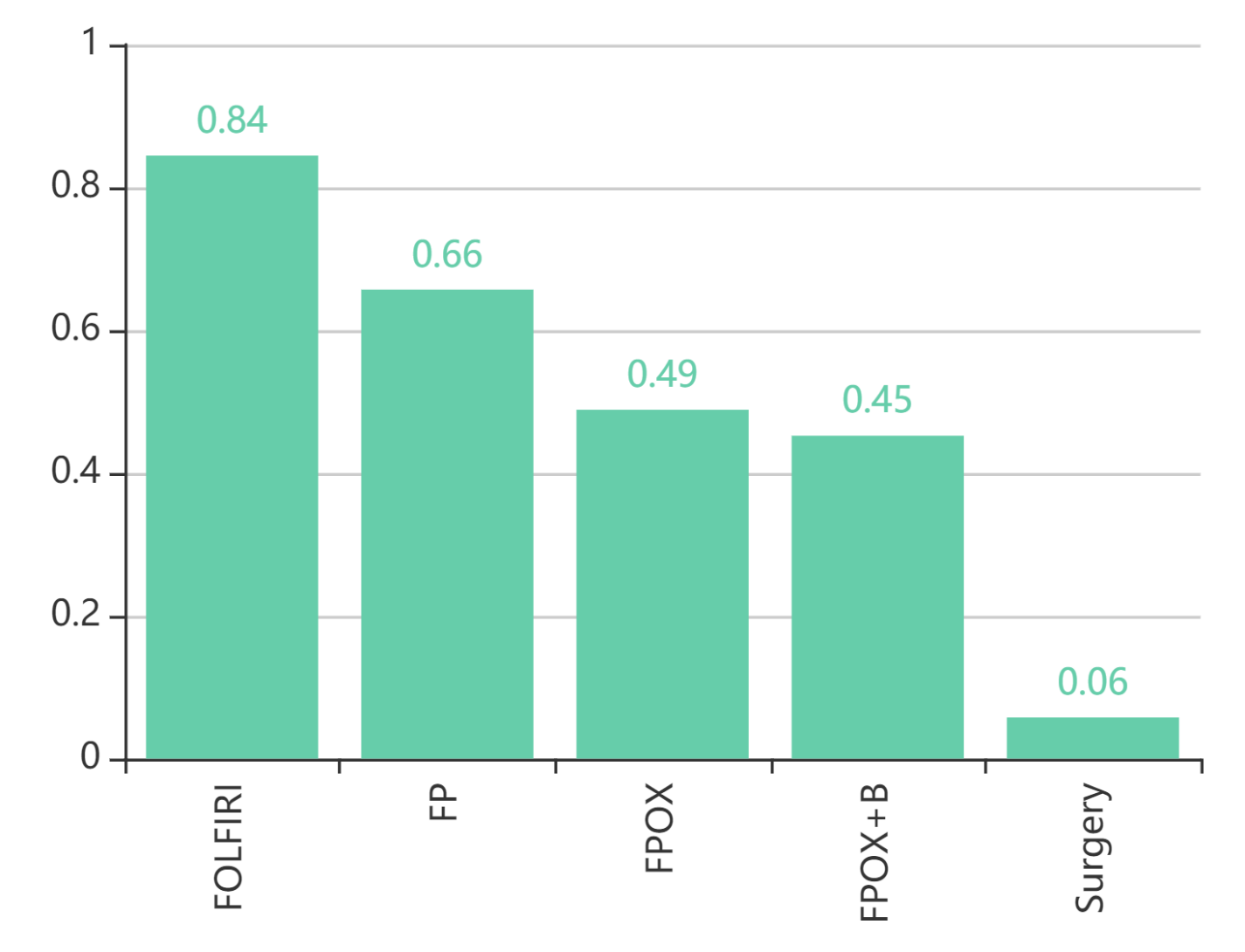


Legend: FPOX: fluoropyrimidine + oxaliplatin, FP: fluoropyrimidine alone, B: bevacizumab, FOLFIRI: infusional fluorouracil + irinotecan

eFigure 3: : Ranking of interventions for overall survival


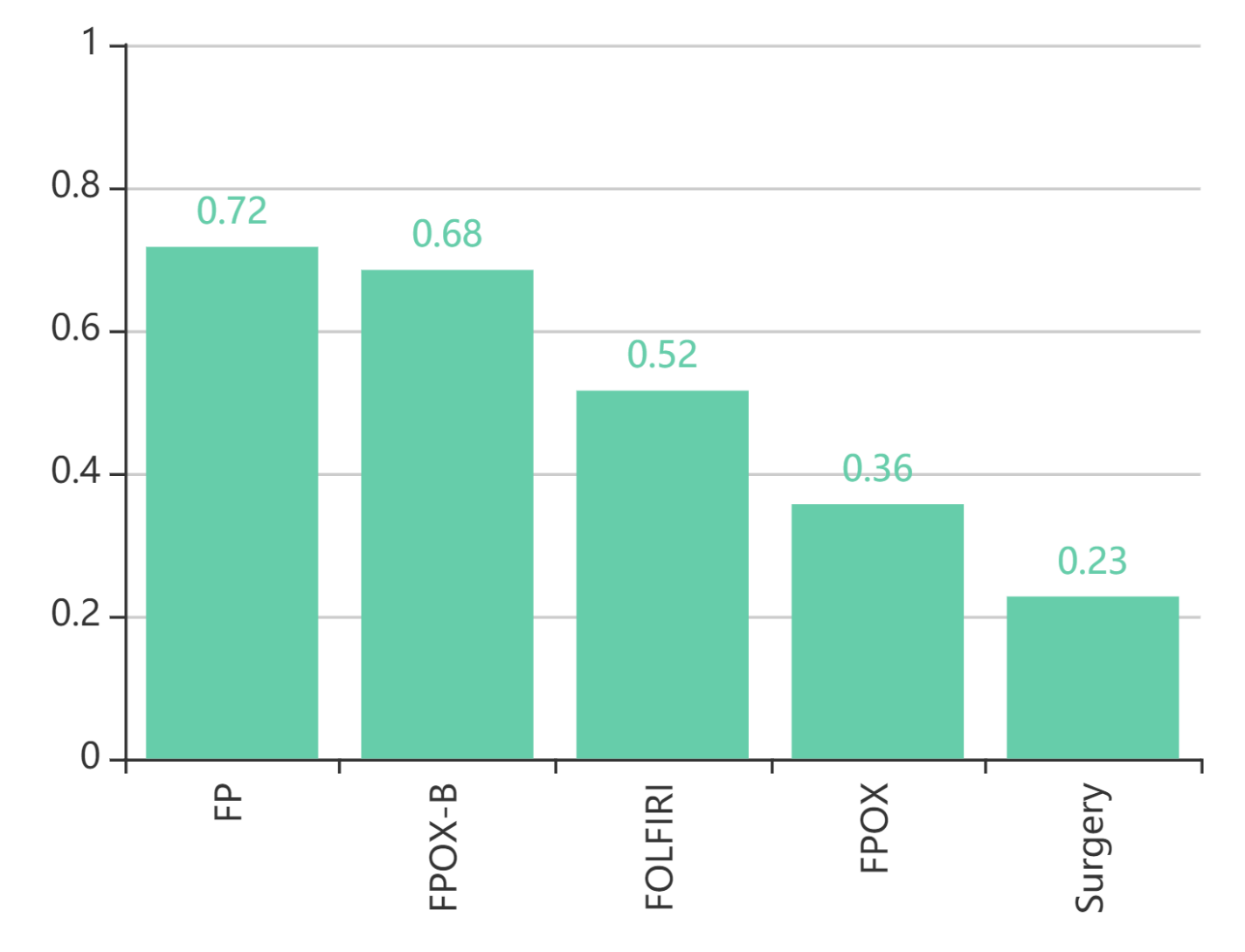


Legend: FPOX: fluoropyrimidine + oxaliplatin, FP: fluoropyrimidine alone, B: bevacizumab, FOLFIRI: infusional fluorouracil + irinotecan

eFigure 4. Risk of bias graph for first-line studies: review authors' judgements about each risk of bias item presented as percentages across all included studies


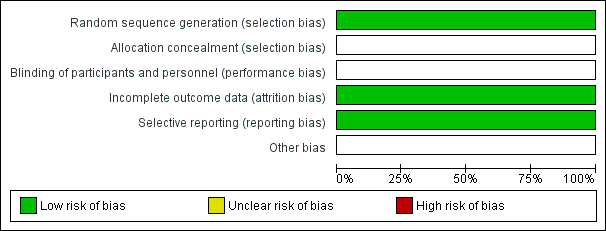


eTable2. Certainty of Evidence Table (GRADE)

| **Certainty assessment** | | | | | | | **Effect** | **Certainty** | **Importance** |
| --- | --- | --- | --- | --- | --- | --- | --- | --- | --- |
| **№ of studies** | **Study design** | **Risk of bias** | **Inconsistency** | **Indirectness** | **Imprecision** | **Other considerations** | **HR*** |  |  |
|  |  |  |  |  |  |  | **(95% CI)** |  |  |
| **DFS - Chemotherapy+Surgery vs Surgery alone** | | | | | | | | | |
| 4 | Randomized trials | not serious | not serious | not serious | not serious | none | **HR 0.73** (0.63-0.84) | ⨁⨁⨁⨁  HIGH | IMPORTANT |
| **DFS – FOLFIRI+Surgery vs Surgery also** | | | | | | | | | |
| Network meta-analysis | Randomized trials | not serious | not serious | not serious | not serious | none | **HR 0.61**  (0.42-0.88) | ⨁⨁⨁⨁  HIGH | IMPORTANT |
| **DFS – FP+Surgery vs Surgery also** | | | | | | | | | |
| Network meta-analysis | Randomized trials | not serious | not serious | not serious | not serious | none | **HR 0.68**  (0.54-0.86) | ⨁⨁⨁⨁  HIGH | IMPORTANT |
| **DFS – FPOX+Surgery vs Surgery also** | | | | | | | | | |
| Network meta-analysis | Randomized trials | not serious | not serious | not serious | not serious | none | **HR 0.0.79**  (0.42-1.47) | ⨁⨁⨁⨁  HIGH | IMPORTANT |
| **OS – FPOX-B+Surgery vs Surgery also** | | | | | | | | | |
| Network meta-analysis | Randomized trials | not serious | not serious | not serious | serious | none | **HR 0.67**  (0.21-2.15) | ⨁⨁⨁◯  MODERATE | IMPORTANT |
| **OS – Chemotherapy+Surgery vs Surgery alone** | | | | | | | | | |
| 4 | Randomized trials | not serious | not serious | not serious | serious | none | **HR 0.88** (0.74-1.05) | ⨁⨁⨁◯  MODERATE | IMPORTANT |
| **OS – FOLFIRI+Surgery vs Surgery also** | | | | | | | | | |
| Network meta-analysis | Randomized trials | not serious | not serious | not serious | serious | none | **HR 0.86**  (0.53-1.41) | ⨁⨁⨁◯  MODERATE | IMPORTANT |
| **OS – FP+Surgery vs Surgery also** | | | | | | | | | |
| Network meta-analysis | Randomized trials | not serious | not serious | not serious | Serious | none | **HR 0.79**  (0.60-1.03) | ⨁⨁⨁◯  MODERATE | IMPORTANT |
| **OS – FPOX+Surgery vs Surgery also** | | | | | | | | | |
| Network meta-analysis | Randomized trials | not serious | not serious | not serious | Serious | none | **HR 0.95**  (0.76-1.20) | ⨁⨁⨁◯  MODERATE | IMPORTANT |
| **OS – FPOX-B+Surgery vs Surgery also** | | | | | | | | | |
| Network meta-analysis | Randomized trials | not serious | not serious | not serious | Serious | none | **HR 0.67**  (0.21-2.15) | ⨁⨁⨁◯  MODERATE | IMPORTANT |

Legend: FPOX: fluoropyrimidine + oxaliplatin, FP: fluoropyrimidine alone, B: bevacizumab, FOLFIRI: infusional fluorouracil + irinotecan
